# Negative chronotropic and inotropic effects of lubiprostone on iPS cell-derived cardiomyocytes via activation of CFTR

**DOI:** 10.1186/s12906-020-02923-6

**Published:** 2020-04-19

**Authors:** Hiraku Akita, Susumu Yoshie, Takafumi Ishida, Yasuchika Takeishi, Akihiro Hazama

**Affiliations:** 1 Department of Cardiovascular Medicine, Fukushima Medical University, Fukushima, Japan; 2 Department of Cellular and Integrative Physiology, School of Medicine, Fukushima Medical University, 1 Hikarigaoka, Fukushima City, 960-1295 Japan

**Keywords:** iPS cells, iPS-CMs, Lubiprostone, ClC-2, CFTR

## Abstract

**Background:**

Lubiprostone (LBP) is a novel chloride channel opener that has been reported to activate chloride channel protein 2 (ClC-2) and cystic fibrosis transmembrane conductance regulator (CFTR). LBP facilitates fluid secretion by activating CFTR in the intestine and is used as a drug for treating chronic constipation. While ClC-2 and CFTR expression has been confirmed in cardiomyocytes (CMs), the effect of LBP on CMs has not yet been investigated. Thus, the present study aimed to investigate the effect of LBP on CMs using mouse-induced pluripotent stem (iPS) cell-derived CMs (iPS-CMs).

**Methods:**

We induced mouse iPS cells into CMs through embryoid body (EB) formation. We compared the differentiated cells to CMs isolated from adult and fetal mice using gene expression, spontaneous beating rate, and contraction ratio analyses.

**Results:**

Gene expression analysis revealed that, in the iPS-CMs, the mRNA expression of the undifferentiated cell markers *Rex1* and *Nanog* decreased, whereas the expression of the unique cardiomyocyte markers cardiac troponin I *(cTnI*) and cardiac troponin T *(cTNT)*, increased. Immunostaining showed that the localization of cTnI and connexin-43 in the iPS-CMs was similar to that in the primary fetal CMs (FCMs) and adult CMs (ACMs). LBP decreased the spontaneous beating rate of the iPS-CMs and FCMs, and decreased the contraction ratio of the iPS-CMs and ACMs. The reduction in the beating rate and contraction ratio caused by LBP was inhibited by glycine hydrazide (GlyH), which is a CFTR inhibitor.

**Conclusion:**

These results suggest that LBP stimulates CFTR in CMs and that LBP has negative chronotropic and inotropic effects on CMs. LBP may be useful for treating cardiac diseases such as heart failure, ischemia, and arrhythmia.

## Background

Lubiprostone (LBP) is an activator of the chloride channel protein 2 (ClC-2) and cystic fibrosis transmembrane conductance regulator (CFTR) channels. In the intestinal epithelium, LBP promotes intracellular Cl^−^ and fluid secretion to the intestinal tract through the activation of CFTR due to prostanoid receptor signaling; therefore, LBP is used as a chronic constipation drug [1]. In murine nasal airway epithelia, LBP independently activates both ClC-2 and CFTR [2, 3]. Furthermore, LBP has been reported to increase the contraction of smooth muscle through the prostaglandin E receptor 1 signaling pathway in the small intestine [4]; however, the effect of LBP on cardiomyocytes (CMs) has not yet been determined.

Cation channels, such as sodium, potassium, and calcium channels in CMs have been well studied, and antiarrhythmic or cardiotonic drugs have been developed that modulate these channel activities [5–7]. Although the function and role of anion channels have also been investigated in CMs using knockout mice, the function and role of chloride channels have not been as extensively investigated as those of other anion channels. For example, it has been previously reported that the role of the ClC-2 channel is to control the activation of cardiac pacemaker and heart rate under pathological conditions [8]. Additionally, it is well known that CFTR, which is also a Cl^−^ channel, is expressed in CMs and involved in cystic fibrosis. A previous study reported that the infarct size induced by ischemia/reperfusion injury of the heart in CFTR knockout mice was larger than that in wild-type mice, and that the cause was loss of cell volume regulation due to CFTR dysfunction [9]. However, the role of ClC-2 and CFTR in CMs has not yet been fully elucidated, since it is difficult to create, purchase, and reproduce genetically manipulated mice such as knockout mice.

iPS cells can be reprogrammed by the effect of several stemness factors, such as Oct3/4, Nanog, Sox2, Klf4, c-Myc, and Lin28 [10–12], and have the ability of self-renewal and pluripotency. Thus, they are similar to embryonic stem (ES) cells, which are isolated from the inner cell mass of a blastocyst [13, 14]. ES cells can be created by destroying the early embryo, and raise ethical issues. Furthermore, cells differentiated from ES cells may cause rejection after cell transplantation. Therefore, the use of iPS cells generated from individual somatic cells can overcome the disadvantages of using ES cells; iPS cells are also expected to be used as powerful tools for regenerative medicine to repair damaged tissues [11]. Furthermore, genomic integration-free iPS cells produced using episomal vectors [15], Sendai viruses [16], or the piggyBac system [17] are regarded as safer because they avoid the risk of tumor formation. iPS cells can be differentiated into various cell types under the appropriate conditions, and CMs differentiated from iPS cells are effective tools not only for cell and tissue replacement, but also for pharmacological and toxicological testing. The use of self-derived iPS cells also does not cause differences in drug effects among individuals due to genetic single nucleotide polymorphisms (SNPs). For these reasons, iPS-CMs are considered to be an effective source of cells for studying the effects of LBP on CMs and the roles of ClC-2 and CFTR. However, to accurately demonstrate the effects of LBP on CMs using mouse iPS-CMs, iPS-CMs must be compared with primary CMs isolated from mouse hearts. Many researchers have reported that the characteristics of cells differentiated from mouse iPS cells are similar to those of primary cultured cells isolated from mice, and they have evaluated the pharmacological action of drugs on both types of cells [18–23]. For example, Kuzmenkin et al. reported that CMs generated from mouse iPS cells have the same functional properties as in vivo CMs. In their study, mouse iPS cells were differentiated into CMs in vitro and compared with primary CMs isolated from mouse hearts using immunocytochemistry, electrophysiology, and drug response. The characteristics of the iPS-CMs were similar to those of the primary CMs, and the response of the iPS-CMs treated with several drugs, such as the Na^+^ channel blocker lidocaine and the Ca^2+^ channel blocker nifedipine, was comparable to that of the primary CMs [18]. Sancho-Bru et al. induced mouse iPS cells into hepatocytes and examined the gene expression and functional characteristics of mouse iPS cell-derived hepatocytes by comparing them with those of primary hepatocytes isolated from mouse livers [20]. Yoshie et al. investigated whether mouse iPS cell-derived airway epithelial cells have the characteristics of native airway epithelial cells by comparing their ciliary beating frequency with that of native airway epithelial cells isolated from mouse airways [22].

The aim of the current study was to examine the effect of LBP on CMs using mouse iPS-CMs and CMs isolated from fetal and adult mouse hearts.

## Methods

### Animals

The present study was performed following the approval of the Animal Care and Use Committee in accordance with the Guidelines for Animal Experiments of Fukushima Medical University (Approval No. 30052). Nine- to 12-week-old C57BL/6 J mice (25–28 g) (CLEA Japan, Tokyo, Japan) and BALB/cAJc1 mice (CLEA Japan) that had been pregnant for 17 days (29–34 g) were used in this study. There were 12 mice in each group. The mice had free access to food and water and were maintained in a temperature- (24–25 °C) and humidity- (50%) controlled room. Animals were also subjected to a 12-h light/dark cycle.

### Mouse iPS cell culture

The culture of mouse iPS cells (20D17), carrying Nanog promoter-driven GFP/IRES/puromycin-resistant gene, was performed as described previously [24]. Briefly, mouse iPS cells were cultured on mouse primary embryonic fibroblast feeder cells (KBL9284400, Oriental Yeast Co. Ltd., Tokyo, Japan) in Dulbecco’s Modified Eagle Medium (DMEM; FUJIFILM Wako Pure Chemical Corporation, Osaka, Japan) containing 15% fetal bovine serum (FBS; Thermo Fisher Scientific Inc., Waltham, MA, USA), 2 mM L-glutamine (FUJIFILM Wako Pure Chemical Corporation), 100 μM non-essential amino acids (FUJIFILM Wako Pure Chemical Corporation), 100 μM 2-mercaptoethanol (2-ME; Thermo Fisher Scientific), 10^3^ units/mL of leukemia inhibitory factor (FUJIFILM Wako Pure Chemical Corporation), and penicillin-streptomycin (FUJIFILM Wako Pure Chemical Corporation).

### Generation of CMs from iPS cells

Differentiation of CMs from mouse iPS cells was performed as previously described [25]. Mouse iPS cells were treated with 0.25% trypsin (FUJIFILM Wako Pure Chemical Corporation) and dispersed into a single-cell suspension in embryoid body (EB) medium, which consisted of knockout DMEM (Thermo Fisher Scientific), 20% FBS, 2 mM L-glutamine, 100 μM non-essential amino acids, 100 μM 2-ME, and penicillin-streptomycin. Next, the cells were cultured in a 24-well plate to allow EB formation using the hanging drop method. After 5 days, the formed EBs were transferred to a gelatin-coated 24-well plate (Greiner Bio-One Co. Ltd., Tokyo, Japan) and cultured in EB medium. The EB medium was exchanged every 2 days.

### Reverse transcription polymerase chain reaction

Total RNA was isolated using an RNeasy Mini kit (Qiagen, Hilden, Germany) according to the manufacturer’s protocol. For cDNA synthesis, Superscript Reverse Transcriptase (TaKaRa Bio Inc., Shiga, Japan) was used, and PCR was performed using Ex Taq (TaKaRa Bio Inc.). The PCR cycling conditions were as follows: 30 or 35 cycles at 94 °C for 30 s, annealing at the temperatures specified for each primer set for 30 s; 72 °C for 30 s; and a final cycle at 72 °C for 7 min. The primer sets and annealing temperatures are shown in Table 1.

**Table 1:** Primer sequences for RT-PCR

Gene	Accession	Sense primer sequence (5'-3')	Antisense primer sequence (5'-3')	Annealing temperature[°C]
Rex1	NM_009556	ACGAGTGGCAGTTTCTTCTTGGGA	TATGACTCACTTCCAGGGGGCACT	65
Nanog	AB903574	GCTTACAAGGGTCTGCTACT	CCTCAGGACTTGAGAGCTTT	60
GFP	NC_011521	AGAAGAACGGCATCAAGGTG	CTCGTTGGGGTCTTTGCTCA	65
GATA4	NC_000080	GCAGCAGCAGTGAAGAGATG	GCGATGTCTGAGTGACAGGA	60
cTn I	NC_000073	CTCCTCTGCCAACTACCGAG	CTCAAACTTTTTCTTGCGGC	60
cTn T	NC_000067	ATCCCCGATGGA	TTCCCACGAGTTTTGGAGAC	65
Cx43	NC_000076	TTGACTTCAGCCTCCAAGG	AATGAACAGCACCGACAGC	65
CFTR	NM_021050	AGTTTCCTGGACAGCTCA	CTAATGGCCTGCTGGAAGAT	60
CLC2	NM_009900	CTTAGAGTGGGAAGAACA	CTCCTTTAGGGTGACAATCC	60
βactin	NM_007614	TTCCTTCTTGGGTATGGAAT	GAGCAATGATCTTGATCTTC	60

### Immunocytochemistry

Mouse iPS-CMs, FCMs, and CMs were immersed in 4% paraformaldehyde (PFA) for 30 min at room temperature, and then washed with phosphate buffered saline (PBS). The cells were permeabilized with 0.1% Triton X-100 for 30 min and then washed with PBS. The cells were then treated with 4% Block Ace Powder (DS Pharma Biomedical Co., Ltd., Osaka, Japan) in PBS for 30 min, followed by staining via incubation at 4 °C overnight with specific antibodies against cardiac troponin I (cTnI) and connexin 43 (Cx43) (Abcam plc., Cambridge, UK). Following this incubation, the cells were incubated with Alexa Fluor 488, 568, and DAPI (Molecular Probes, Eugene, USA). Fluorescent images were captured with a confocal laser scanning microscope A1R (Nikon Instech, Tokyo, Japan).

### Isolation and culture of CMs from adult mouse hearts

The CMs of adult mice were isolated using the Langendorff-free method, as described in a previous study [26]. The buffers and medium for the isolation and culture of the CMs were also prepared according to the same report. In brief, the mice were euthanized with an anesthetic containing 5 mg/kg butorphanol, 0.3 mg/kg medetomidine, and 4 mg/kg midazolam, after which their chests were opened. EDTA buffer, which consisted of 130 mM NaCl, 5 mM KCl, 0.5 mM NaH_2_PO_4_, 10 mM HEPES, 10 mM glucose, 10 mM BDM, 10 mM taurine, and 5 mM EDTA, was injected into the right ventricle, following which the heart was excised and placed in a 60 mm dish. EDTA buffer, perfusion buffer (130 mM NaCl, 5 mM KCl, 0.5 mM NaH_2_PO_4_, 10 mM HEPES, 10 mM glucose, 10 mM BDM, 10 mM taurine, and 1 mM MgCl_2_), and collagenase buffer (0.5 mM collagenase 2, 0.5 mM collagenase 4, and protease XIV) were continuously injected into the left ventricle, and then the heart tissues were dissociated by gentle pipetting. Next, stop buffer, which consisted of perfusion buffer containing 5% FBS, was added to the 60 mm dish, and the cell suspension was transferred to a 50 mL tube through a 100 μm cell strainer. After the dissociated CMs were gradually exposed to calcium, they were plated and cultured on a cell culture dish. The culture medium was changed every 2 days.

### Isolation and culture of FCMs from fetal mouse hearts

Isolation of FCMs was performed according to a previous study [27]. Briefly, 17-day pregnant BALB/cAJc1 mice (CLEA Japan) were euthanized by cervical dislocation, and the uterus was isolated. Euthanasia by cervical dislocation has been widely used in animal experiment, since it can cause a fast and painless death and avoid damage to the fetus [27–34]. Furthermore, it does not chemically contaminate tissues. The excised uterus was then washed with PBS containing penicillin-streptomycin, followed by removal of the fetus. The heart was removed from the fetus, washed with PBS, then minced and incubated in PBS containing 0.05% trypsin for 30 min at 37 °C. The digested heart tissue was homogenized by vortexing and then placed into a 15 mL tube containing culture medium, which consisted of DMEM with 10% FBS and penicillin streptomycin. The 15 mL tube was centrifuged, and the cell pellet was suspended in culture medium. The cell suspension was then plated and cultured on a cell culture dish, and the culture medium was changed every 2 days.

### Analysis of beating rate

The beating rates of the iPS-CMs and FCMs were analyzed using several chemical compounds. Isoproterenol ISP (ISP) (0.4, 4, 40, and 400 nM) (Tokyo Chemical Industry Co., Ltd., Tokyo, Japan), nitrendipine (NDP) (10 μM) (Wako), LBP (0.05, 0.5, and 5 μM) (Toronto Research Chemicals, North York, Canada), glycine hydrazide (GlyH) (5 μM) (Santa Cruz Biotechnology, Inc., Heidelberg, Germany), and/or CdCl_2_ (10 μM) (Wako) were added to the culture medium. The iPS-CMs and FCMs treated with these chemical compounds were incubated for 20 min at 37 °C in a 5% CO_2_ incubator, and beating of the iPS-CMs and FCMs was captured at 16 frames per second using an ORCA-ER EMCCD camera (Hamamatsu Photonics K.K., Shizuoka, Japan) connected to an IX-71 microscope (Olympus Corporation, Tokyo, Japan). Changes in beats per minute (bpm) were then analyzed.

### Analysis of the contraction ratio

The ACMs treated with ISP, LBP, GlyH, or CdCl_2_ were stimulated with monophasic pulses at 2 to 5 V/cm and a frequency of 0.5 Hz with pulse durations of 20 ms using an electronic stimulator 3F46 (Nippon Avionics Co., Tokyo, Japan). The contraction of iPS-CMs and ACMs was captured at 16 frames per second with an ORCA-ER EMCCD camera connected to an IX-71 microscope. The difference between the relaxation and contraction lengths (ΔL) of each iPS-CM and ACM was analyzed using ImageJ (US National Institutes of Health, Bethesda, USA) [35], and the ratio of ΔL to relaxation length was analyzed to determine the contraction ratio.

### Statistical analysis

All statistical analyses were performed with EZR (Saitama Medical Center, Jichi Medical University, Saitama, Japan), which is a graphical user interface for R (The R Foundation for Statistical Computing, Vienna, Austria) [36]. All experiments were performed independently at least five times. Data are expressed as the mean ± SD. All statistical significance was verified using ANOVA and Wilcoxon signed-rank sum tests. *P* values of < 0.05 were considered statistically significant.

## Results

### Analysis of the iPS-CMs characteristics

**Fig. 1:**
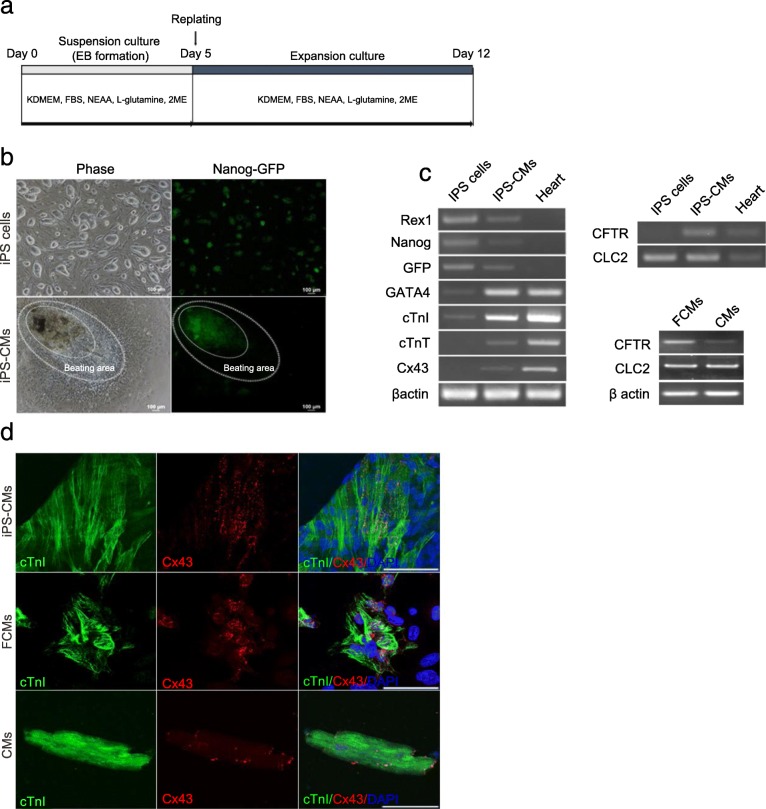
Generation of cardiomyocytes from iPS cells. **a**: Time course for the generation of cardiomyocytes (CMs). **b**: Undifferentiated mouse iPS cells and iPS-CMs. Left panel: phase contrast Right panel: Nanog-promoter-driven GFP. Bars indicate 100 μm. **c**: RT-PCR analysis of gene expression in undifferentiated iPS cells, iPS-CMs, and the heart, using specific primers to identify undifferentiated markers, CM markers, chloride channel markers, and a ubiquitous housekeeping gene, β-actin, as indicated in the left column. **d**: Immunocytochemistry of iPS-CMs, FCMs, and ACMs using cTnI and Cx43 antibodies. Bars indicate 50 μm

We induced iPS cells into CMs through EB formation (Fig. 1a). To confirm the state of the iPS-CMs, a Nanog-GFP reporter system was used as an efficient marker to mimic endogenous Nanog gene expression. Undifferentiated iPS cells showed GFP expression, which is controlled by the Nanog promoter; however, GFP expression was not observed in the beating region of iPS-CMs (Fig. 1b). We further investigated whether the expression of cardiomyocyte markers could be detected in iPS-CMs. The CM-specific markers *GATA4*, *cTnI*, cardiac troponin T (*cTnT*), and *Cx43* were expressed in iPS-CMs. Additionally, the expression of *ClC-2* and *CFTR* could be detected in iPS-CMs, FCMs, and ACMs. Conversely, the expression of the undifferentiated markers *Rex1*, *Nanog*, and *GFP* was weak in the iPS-CMs compared to that in the iPS cells (Fig. 1c). Next, to precisely examine whether iPS-CMs expressed CM markers, these cells were evaluated by double immunostaining. cTnI- and Cx43-positive cells were clearly observed in the iPS-CMs, FCMs, and ACMs (Fig. 1d). These results suggest that iPS-CMs have the characteristics of CMs.

### Analysis of iPS-CMs and FCMs beating after LBP treatment

**Fig. 2:**
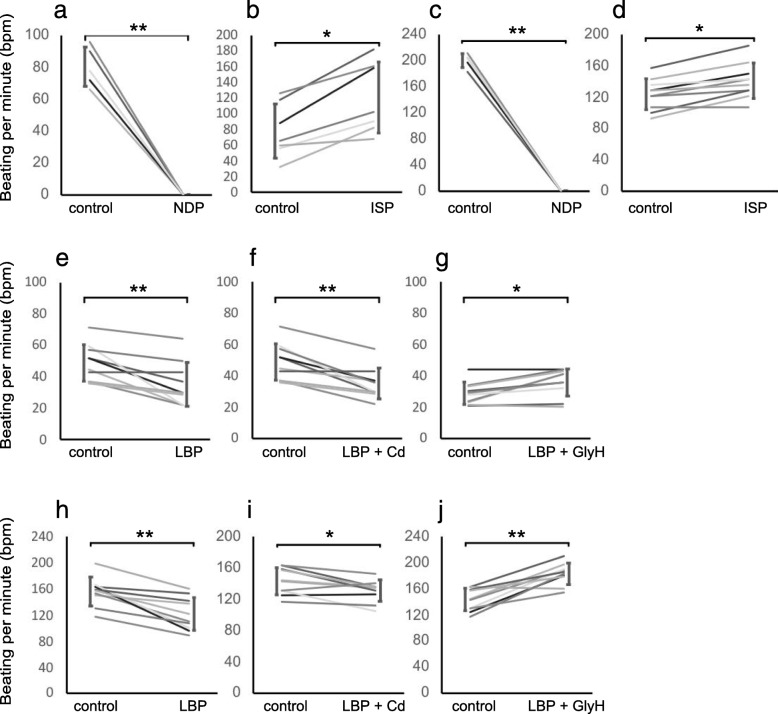
Beating response to chemical compounds. **a** and **b**: Changes in the beating rate of iPS-CMs treated with 10 μM NDP (**a**) (*n* = 5) and 400 nM ISP (**b**) (*n* = 7). **c** and **d**: Changes in the beating rate of FCMs treated with 10 μM NDP (**c**) (*n* = 5) and 400 nM ISP (**d**) (*n* = 7). **e**–**g**: Changes in the beating rate of iPS-CMs treated with 5 μM LBP, 10 μM CdCl_2_, or 5 μM GlyH (*n* = 10). **h**–**j**: Changes in the beating rate of FCMs treated with 5 μM LBP, 10 μM CdCl_2_, or 5 μM GlyH (*n* = 10). Data are expressed as the mean ± SD. (**P < 0.01 and *P < 0.05 vs. control)

First, CMs were evaluated for their responses to NDP, which is a calcium channel blocker, and ISP, which is a β-adrenergic agonist. However, almost all of the ACMs isolated from the adult hearts did not exhibit spontaneous beating. Therefore, beating analysis was performed using iPS-CMs and FCMs. Spontaneous beating of the iPS-CMs and FCMs treated with 10 μM NDP stopped (bpm was 0) (Fig. 2a and c). However, the bpm of the iPS-CMs treated with 400 nM ISP increased from 78.0 ± 34.3 to 120 ± 45.2 (Fig. 2b). Additionally, the bpm of the FCMs treated with 400 nM ISP increased from 123 ± 19.7 to 141 ± 22.4 (Fig. 2d). Next, the effect of LBP on spontaneous beating was examined. The bpm of the iPS-CMs treated with 5 μM LBP decreased from 48.8 ± 7.1 to 34.5 ± 13.9 (Fig. 2e). To determine which chloride channels might contribute to the reduction of the beating rate, we examined the beating rate using CdCl_2_, a ClC-2 channel blocker [37], and GlyH, a CFTR channel blocker [38]. While the bpm of the iPS-CMs treated with 5 μM LBP and 10 μM CdCl_2_ did not change (Fig. 2f), that of the iPS-CMs treated with 5 μM LBP and 5 μM GlyH slightly increased from 28.8 ± 11.6 to 35.8 ± 8.7 (Fig. 2g). When the FCMs were also treated with the same chemical compounds, the changes in bpm were similar to those of the iPS-CMs (Fig. 2h-j). These results indicate that LBP decreased bpm not through the ClC-2 channel, but through CFTR.

### Contraction analysis of iPS-CMs and ACMs after LBP treatment

**Fig. 3:**
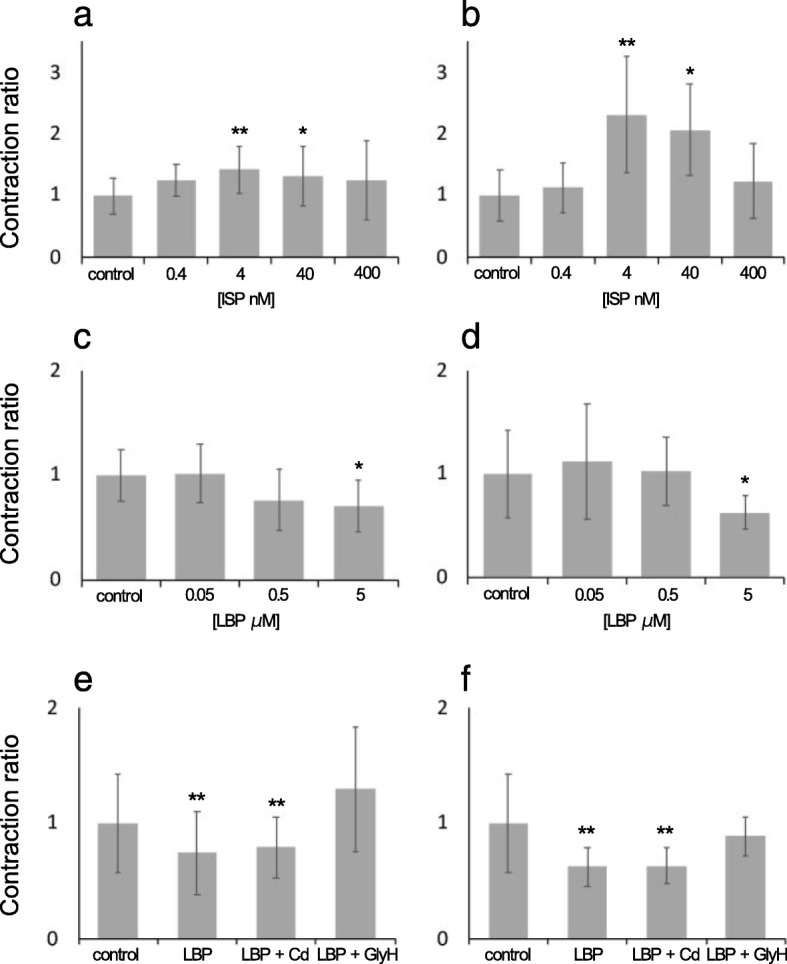
Contraction response to chemical compounds**. a** and **b**: Changes in the contraction ratio of iPS-CMs (**a**) and ACMs (**b**) treated with ISP at various concentrations. **c** and **d**: Changes in the contraction ratio of iPS-CMs (**c**) and ACMs (**d**) treated with various concentrations of LBP. **e** and **f**: Changes in the contraction ratio of iPS-CMs (**e**) and ACMs (**f**) treated with 5 μM LBP, 10 μM CdCl_2_, or 5 μM GlyH. Data are expressed as the mean ± SD. (*n* = 10, **P < 0.01 and *P < 0.05 vs. control)

**Fig. 4:**
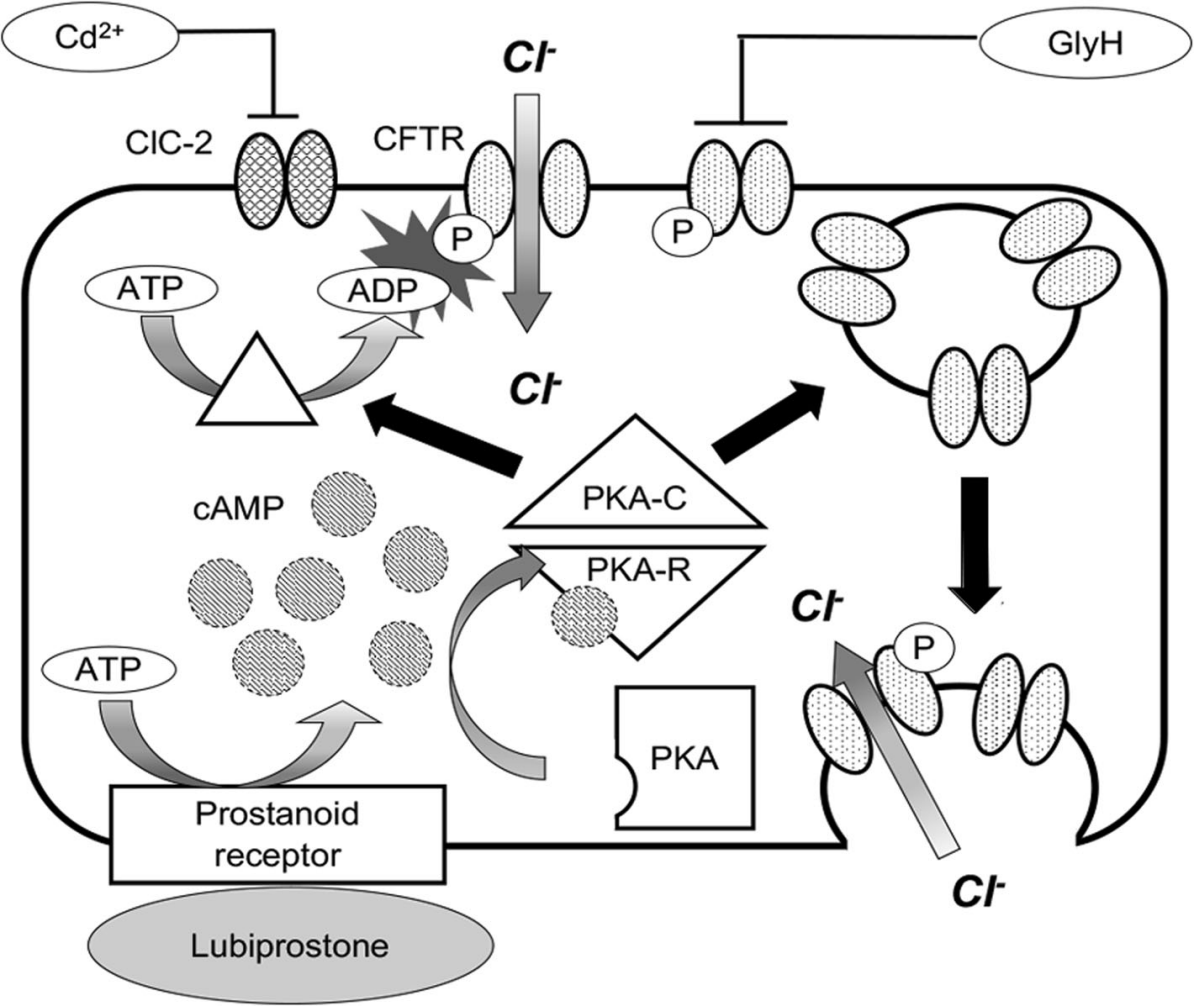
Mechanisms of lubiprostone action on iPS-CMs. Lubiprostone decreased the beating rate and contraction ratio of iPS-CMs due to the activation of CFTR and the increase in CFTR protein at the plasma membrane though prostanoid receptor signaling

The contraction of iPS-CMs and ACMs was investigated. Since FCMs tightly adhere to the cell culture dish, their relaxation and contraction lengths could not be accurately measured. First, the contraction ratio of the iPS-CMs treated with ISP at various concentrations was investigated. The contraction ratio of the iPS-CMs significantly increased in the presence of 4 nM or 40 nM ISP (Fig. 3a). Similarly, the contraction rate of the ACMs treated with 4 nM or 40 nM ISP was higher, but decreased at 400 nM ISP (Fig. 3b). Next, the effect of LBP on the contraction ratio was examined. The contraction ratio of the iPS-CMs and ACMs treated with 5 μM LBP significantly decreased compared to that of the control (Fig. 3c and d). We found that CdCl_2_ did not inhibit the contraction ratio of the iPS-CMs and ACMs treated with LBP (Fig. 3e), although GlyH did inhibit the reduction in the contraction ratio (Fig. 3f). These results indicate that LBP decreased the contraction ratio not through the ClC-2 channel, but through CFTR (Fig. 4).

## Discussion

In the present study, we demonstrated that LBP decreased the beating rate and contraction ratio of iPS-CMs, FCMs, and ACMs through CFTR, but not CIC-2. While several research groups have reported that LBP activates CFTR and ClC-2 in the intestinal and nasal airway epithelium [1–3], there have been no studies on the roles of LBP in CMs. To the best of our knowledge, the present study is the first to focus on the effect of LBP on CMs.

First, we induced iPS cells into CMs according to a previously-described simple protocol [25]. GATA4 is expressed in the early fetal heart, and is known to regulate the expression of several genes, such as atrial natriuretic peptide, brain-type natriuretic peptide, and α-type myosin heavy chain [39, 40]. Thus, GATA4 is an important factor in cardiac development, and is a specific marker of CM differentiation [41]. Additionally, cTnI and cTnT, which are present in myocardial fibers, are components of the troponin complex, and they control the contraction of CMs in response to changes in calcium ion concentration. Moreover, gap junctions formed from the connexin family have the function of synchronizing cardiac contraction with adjacent CMs. Cx43, which is a member of the connexin family, is the major protein expressed in the mammalian heart [42]. These markers are often used to evaluate the differentiation of CMs from iPS cells [43–46]. Therefore, we examined the expression of *GATA4*, *cTnI, cTnT*, and *Cx43* mRNA by RT-PCR and the expression of the corresponding proteins by immunostaining*.* The expression of these genes could be successfully detected in the iPS-CMs, and the cTnI and Cx43 proteins were localized to the appropriate region (Fig. 1c and d). These results indicate that iPS-CMs have the characteristics of native CMs.

The calcium channel blocker NDP inhibits increases in intracellular calcium concentration. ISP stimulates β-adrenergic receptors on CMs and increases the beating rate due to the increase in intracellular cyclic adenosine monophosphate (cAMP) and calcium ion concentration [47, 48]. Therefore, we analyzed whether iPS-CMs respond to these chemical compounds. NDP suppressed the beating of iPS-CMs, and ISP increased the beating rate (Fig. 2a and b). It has been reported that ISP increases not only the beating rate but also the contraction force [47, 48], and in the current study, the contraction ratio of iPS-CMs treated with ISP was also increased (Fig. 3a). The changes in the beating rate and contraction ratio of the FCMs and ACMs treated with these compounds were also similar to those of the iPS-CMs. The response of CMs treated with these chemical compounds is consistent with that reported in previous studies [49–51]. Therefore, our results indicate that iPS-CMs have the physiological functional characteristics of native CMs.

The beating rate and contraction ratio of iPS-CMs, FCMs, and ACMs decreased with LBP treatment (Figs. 2e, h, 3c, and d). These results are not consistent with those of a previous study [8] that used anti-ClC-2 antibody or ClC-2 knockout mice. The activation of ClC-2 in the heart has been reported to play important roles in the diastolic depolarization and firing of pacemaker cells [8]. However, LBP is known to activate not only ClC-2 but also CFTR in the nasal airway epithelium [2, 3]. Thus, we analyzed the beating rate and contraction ratio after treatment with CdCl_2_ and GlyH to investigate which chloride channels contributed to the reduction of the beating rate and contraction ratio. The CFTR blocker GlyH inhibited the effect of LBP, while the CLC-2 blocker CdCl_2_ did not (Figs. 2h-j and 3e and f); therefore, GlyH acts as an LBP antagonist. It has been reported that the target chloride channel of LBP is dependent on the tissue type. For example, while LBP activates CFTR on the intestinal epithelium [1], it activates both CFTR and ClC-2 on the nasal epithelium [2, 3]. In the present study, we clarified that LBP activates CFTR in CMs but not ClC-2. In addition, LBP has been reported to increase intracellular cAMP concentration in the intestinal and nasal epithelium. It is well known that an increase in intracellular calcium concentration due to an increase in intracellular cAMP concentration results in an increase in the beating rate and contraction ratio of CMs. However, the results of the current study indicate that the beating rate and contraction ratio of the CMs treated with LBP decreased compared to those of the untreated CMs. On the other hand, LBP has been demonstrated to increase CFTR protein levels at the apical membrane through prostanoid receptor signaling [1, 52]. Thus, we consider that the depolarization and inward Ca^2+^ currents in LBP-treated CMs were disturbed by actively inward flowing Cl^−^ currents mediated by CFTR (Fig. 4). Further investigation is required to elucidate the mechanisms of action of LBP.

## Conclusion

The results of the current study suggest that LBP decreased the beating rate and contraction ratio of iPS-CMs by activating CFTR. Since LBP has negative inotropic and chronotropic effects on FCMs and ACMs, iPS-CMs are considered to be an effective cell source for investigating the effects of chemical compounds and channel function. In a future study, we will examine the effect of LBP on human CMs using human iPS-CMs.

## Data Availability

The datasets used and/or analyzed during the current study are available from the corresponding author upon reasonable request.

## Abbreviations

LBP: Lubiprostone
CLC-2: Chloride channel protein 2
CFTR: Cystic fibrosis transmembrane conductance regulator
CMs: Cardiomyocytes
iPS: Induced pluripotent stem
iPS-CMs: IPS cell-derived cardiomyocytes
EB: Embryoid body
cTnI: Cardiac troponin I
cTnT: Cardiac troponin T
FCMs: Fetal cardiomyocytes
ACMs: Adult cardiomyocytes
GlyH: Glycine hydrazide
ES: Embryonic stem
DMEM: Dulbecco’s Modified Eagle Medium
FBS: Fetal bovine serum
2-ME: 2-mercaptoethanol
PBS: Phosphate buffered saline
Cx43: Connexin 43
ISP: Isoproterenol
NDP: Nitrendipine
bpm: Beats per minute
